# Misclassification and characterization of exposure to humidifier disinfectants using a questionnaire

**DOI:** 10.1186/s12889-021-11459-4

**Published:** 2021-07-27

**Authors:** Hyeonsu Ryu, Yoon-Hyeong Choi, Eunchae Kim, Jinhyeon Park, Seula Lee, Jeonggyo Yoon, Eun-Kyung Jo, Youngtae Choe, Jung Heo, Wonho Yang

**Affiliations:** 1 Department of Occupational Health, Daegu Catholic University, Hayang-eup, Gyeongsan-si, Gyeongbuk 38430 South Korea; 2 Department of Preventive Medicine, Gachon University College of Medicine, Yeonsu-gu, Incheon, 21936 South Korea

**Keywords:** Humidifier disinfectants, Past exposure assessment, Misclassification, Questionnaire

## Abstract

**Background:**

Lung disease caused by exposure to chemical substances such as polyhexamethylene guanidine (PHMG) used in humidifier disinfectants (HDs) has been identified in Korea. Several researchers reported that exposure classification using a questionnaire might not correlate with the clinical severity classes determined through clinical diagnosis. It was asserted that the lack of correlation was due to misclassification in the exposure assessment due to recall bias. We identified the cause of uncertainty to recognize the limitations of differences between exposure assessment and clinical outcomes assumed to be true value. Therefore, it was intended to check the availability of survey using questionnaires and required to reduce misclassification error/bias in exposure assessment.

**Methods:**

HDs exposure assessment was conducted as a face-to-face interview, using a questionnaire. A total of 5245 applicants participated in the exposure assessment survey. The questionnaire included information on sociodemographic and exposure characteristics such as the period, frequency, and daily usage amount of HDs. Based on clinical diagnosis, a 4 × 4 cross-tabulation of exposure and clinical classification was constructed. When the values of the exposure rating minus the clinical class were ≥ 2 and ≤ − 2, we assigned the cases to the overestimation and underestimation groups, respectively.

**Results:**

The sex ratio was similar in the overestimation and underestimation groups. In terms of age, in the overestimation group, 90 subjects (24.7%) were under the age of 10, followed by 52 subjects (14.2%) in their 50s. In the underestimation group, 195 subjects (56.7%) were under the age of 10, followed by 80 subjects (23.3%) in their 30s. The overestimation group may have already recovered and responded excessively due to psychological anxiety or to receive compensation. However, relatively high mortality rates and surrogate responses observed among those under 10 years of age may have resulted in inaccurate exposure in the underestimation group.

**Conclusions:**

HDs exposure assessment using a questionnaire might not correlate with adverse health effects due to recall bias and various other causes such as recovery of injury and psychological anxiety. This study revealed exposure misclassification and characteristics affected by HDs and proposed a questionnaire-based exposure assessment methodology to overcome the limitations of past exposure assessment.

**Supplementary Information:**

The online version contains supplementary material available at 10.1186/s12889-021-11459-4.

## Background

A humidifier may be one of the essential devices required in the dry winter season. Humidifiers emit water vapor or steam to increase humidity. In Korea, most of the homes and offices use humidifiers to maintain indoor humidity and prevent respiratory infections and infections caused by viruses [1]. However, when the water tank of the humidifier is not kept clean, growth of germs, molds, and/or algae can be observed. This results in contaminated vapor, which can enter the lungs directly and cause risk to human health [2].

To prevent the propagation of germs in a humidifier, humidifier disinfectants (HDs) were designed as a chemical additive in Korea. According to the Korea Centers for Disease Control and Prevention (KCDC), approximately 20 different products have been sold since 1994. However, these were completely banned in 2011 when their risk was identified [3]. The major ingredients in the HDs were polyhexamethylene guanidine (PHMG), oligo (2-)ethoxyethoxyethyl guanidine chloride (PGH), chloromethylisothiazolinone (CMIT), and methylisothiazolinone (MIT) [4]. PHMG was the most commonly used ingredient in HDs. These products were manufactured by multinational companies, including Oxy Ssackssak New Gaseupgi Dangbun of Reckitt Benckiser (U.K.), Homeplus Gaseupgi Mate of Tesco (U.K.), Nwith For Humidifier of Medentech (Ireland), Homekeeper Gaseupgi Hanbune Ssak of Henkel (Germany), Sandokkaebi Gaseupgi Punisher of Daiso (Japan), and Vegetable Home Gaseupgi Cleanup of Costco (U.S.) [5].

Epidemiological research and toxicological experiments have shown the effects of exposure to HDs, such as lung disease, asthma, and interstitial lung diseases [6, 7]. By February 2020, a total of 6735 people had enrolled for compensation due to the damage caused by HDs, and the compensation request can be applied through a website [8]. Among them, 1528 died and 487 have had lung disease thus far.

Although HDs have been produced and used in other countries such as the United States of America, Germany, and Japan, they are made up of alcohol or natural ingredients such as eucalyptus; furthermore, they were not widely used. The U.S. Environmental Protection Agency (EPA) recommends that humidifiers should be rinsed with tap water several times after cleaning with a humidifier cleaner or HD to prevent chemicals from spreading through the air [9].

Exposure assessment by personal exposure or biomonitoring was not possible because the adverse effects of HD exposure could not be determined in 2011. Therefore, past exposure assessment could only be estimated using a questionnaire and an indoor air quality model [10]. For clinical classification, HD-related lung damage has been categorized as class 1 (unlikely), class 2 (possible), class 3 (probable), class 4 (definite), and indeterminate through the deliberation of a committee composed of clinicians and public health doctors in an initiative of the Ministry of Environment, Korea [11]. Clinical evidence of HDs exposure included (1) acute or subacute development of cough, dyspnea, or breathlessness, (2) physical signs of spontaneous air leakage, including subcutaneous or mediastinal emphysema, and (3) chest radiographic features of terminal bronchiolar damage [12]. The four classes in the clinical classification were identified by HD-specific singularity, while it might be argued that exposure rating based on the questionnaire may be misclassified due to erroneous statements, leading to recall bias. Therefore, a new methodology was required to reduce misclassification in exposure assessment. This study aimed to propose a methodology to effectively classify the levels of exposure to HDs using a questionnaire.

## Methods

### HD exposure survey

HD exposure assessment survey was conducted face-to-face with by public health experts who qualified by completing regular education from the Korean Society of Environmental Health (KSEH). A total of 25 interviewers from KSEH including authors participated in this survey. Furthermore, the survey results were and cross-checked to enhance the reliability of the questionnaire. They visited the homes of each subject who had applied for compensation and conducted personal interviews and home investigation to complete the questionnaire or checklist related to HD usage. In cases where the person was deceased or unavailable for interview because they were too young under the age of 13, the investigation was conducted with one of the family members who lived with them, such as their parents [13]. At this time, the consent form was obtained through a document with handwritten signature. The questionnaire included information of sociodemographic and exposure characteristics such as the product names of the HD used, ingredients, usage period, frequency of use, and daily usage amount of humidifier disinfectant. The questionnaire used was illustrated in the supplementary material. Additionally, information on the room volume, humidifier location, spraying direction, and objective evidence such as photographs of products and receipt as a proof was collected.

A total of 5245 applicants (4028 survivors and 1217 deaths) completed the exposure survey by the end of 2018. Among them, 3456 used PHMG-containing products. This study was approved by the Institutional Review Board of Daegu Catholic University (IRB No. CUIRB-2016-011).

### Level of exposure to HDs

To assess exposure to HDs in indoor environments, exposure levels, which are a function of indoor concentration and exposure time, were calculated. The ventilation rate for the room used was not considered. The concentration of PHMG dissolved in HD product was used according to the results of the Ministry of Food and Drug Safety [3]. The indoor air concentration of PHMG, cumulative exposure time, and exposure level were calculated using the following equations:

- Indoor air concentration (μg/m^3^) = average daily use (mL) x PHMG concentration in products (μg/mL) / volume of used space (m^3^).
- Cumulative exposure time (hr) = total years x months per year x weeks per month x days per week x hours per day.
- Exposure level (μg/m^3^·hr) = indoor concentration (μg/m^3^) x cumulative exposure time (hr).

Many studies have classified exposure into 4 classes according to the clinical diagnosis criteria for lung diseases [14, 15]. Accordingly, the clinical class and exposure rating were classified into four stages. Clinical classification, which was performed by a committee made up of respiratory pulmonologists, radiologists, pathologists, and epidemiologists without information on the type of HDs, divided HD-related lung damage as class 1 (unlikely), class 2 (possible), class 3 (probable), and class 4 (definite) [16].

The calculated indoor air concentration, cumulative exposure time, and exposure level were arranged in the order of maximum to minimum and then classified by percentage based on clinical diagnosis class that was assumed as the true value [17]. The exposure classification was defined as rating 1 (extreme low exposure), rating 2 (low exposure), rating 3 (medium exposure), and rating 4 (high exposure).

When the value of the exposure rating minus the clinical class was 0 or 1, we assumed these to be the true value. However, when the value was ≥2 or ≤ − 2, we assigned the participants to the overestimation group (high exposure but mild symptoms) or the underestimation group (low exposure but severe symptoms), respectively [18]. The exposure-response relationship was derived from the relationship between exposure to HDs and development of lung disease, excluding the overestimation and underestimation groups [19, 20].

### Statistical analysis

Statistical analyses were performed using SPSS version 19 (IBM Company, USA). A chi-square test was used to analyze the relationship between clinical class and exposure rating. The significance level was set as *p*-value of < 0.05 in all analyses. Using Origin 8.0 (Origin Lab. Co., Northampton, MA, USA), the relationship between clinical class and exposure level was derived by the curve fitting technique.

## Results

Social and demographic characteristics according to clinical classification are shown in Table 1. The total number of subjects in classes 1 and 2 by clinical classification was 4632. The demographic characteristics according to the clinical class groups were significantly different (*p* <  0.01). The number of male and female subjects was similar at 2675 and 2547, respectively, and the number of subjects with unknown sex was relatively very low. The number of survived subjects increased from class 1 to class 4. In terms of age at damage, the number of applicants under the age of 10 was 1536, and 452 subjects had unknown age. There were 339 pregnant women and 439 fetuses among those exposed to HDs.

**Table 1:** Social and demographic characteristics according to clinical classification

Characteristics	Class 1		Class 2		Class 3		Class 4		Indetermination		Total		*p*-value
	N	(%)	N	(%)	N	(%)	N	(%)	N	(%)	N	(%)	
Total	4351	83.0	281	5.4	204	3.9	264	5.0	145	2.8	5245	100.0	
Sex													< 0.01
Male	2285	85.4	113	4.2	89	3.3	146	5.5	70	2.6	2675	100.0	
Female	2063	81.0	168	6.6	115	4.5	118	4.6	55	2.2	2547	100.0	
Unknown	3	13.0	0	0.0	0	0.0	0	0.0	20	87.0	23	100.0	
Survival status													< 0.01
Survival	3479	86.4	215	5.3	133	3.3	72	1.8	72	1.8	4027	100.0	
Death	872	71.6	66	5.4	71	5.8	73	6.0	73	6.0	1218	100.0	
Age at damage (years)													< 0.01
< 10	1088	70.8	142	9.2	106	6.9	145	9.4	55	3.6	1536	100.0	
10s	117	91.4	3	2.3	1	0.8	3	2.3	4	3.1	128	100.0	
20s	299	89.0	10	3.0	8	2.4	12	3.6	7	2.1	336	100.0	
30s	755	82.3	41	4.5	33	3.6	72	7.9	16	1.7	917	100.0	
40s	467	91.2	20	3.9	12	2.3	6	1.2	7	1.4	512	100.0	
50s	501	91.3	18	3.3	14	2.6	8	1.5	8	1.5	549	100.0	
60s	459	91.8	18	3.6	12	2.4	4	0.8	7	1.4	500	100.0	
70s	240	92.7	12	4.6	3	1.2	1	0.4	3	1.2	259	100.0	
≥ 80	53	94.6	1	1.8	0	0.0	0	0.0	2	3.6	56	100.0	
Unknown	372	82.3	16	3.5	15	3.3	13	2.9	36	8.0	452	100.0	
Susceptible group													< 0.01
Pregnant women	261	77.0	10	3.0	18	5.3	44	13.0	6	1.7	339	100.0	
Fetus	311	70.8	42	9.6	30	6.8	17	3.9	39	8.9	439	100.0	
Others	3779	84.6	229	5.1	156	3.5	203	4.6	100	2.2	4467	100.0	

The product names of HDs used according to clinical class are shown in Table 2. The HD products and chemical type according to the clinical classification showed significant differences (*p* <  0.01). Most of the subjects answered that they used the Oxy Ssakssak New Gaseupgi Dangbun, followed by Cefu Gaseupgi Salgyunje in clinical classes 1 and 2, and by Ekyung Gaseupgi Mate in clinical classes 3 and 4. However, 252 subjects did not remember the name of the HD products used in clinical classes 3 and 4. The main ingredient of the HDs used was PHMG.

**Table 2:** Product names of humidifier disinfectants according to clinical classification

	Class 1, 2		Class 3, 4		Indetermination		Total		*p*-value
	N	(%)	N	(%)	N	(%)	N	(%)	
Products	4632	88.3	468	8.9	145	2.8	5245	100.0	
Oxy Ssakssak New Gaseupgi Dangbun	3419	88.2	356	9.2	101	2.6	3876	100.0	< 0.01
Ekyung Gaseupgi Mate	458	92.3	24	4.8	14	2.8	496	100.0	
E-Mart Gaseupgi Salgyunje	112	91.8	9	7.4	1	0.8	122	100.0	
WiseLect Gaseupgi Salgyunje	77	74.0	26	25.0	1	1.0	104	100.0	
Homeplus Gaseupgi Chungjungje	105	89.0	12	10.2	1	0.8	118	100.0	
Vegetable Home Gaseupgi Cleanup	44	97.8	1	2.2	0	0.0	45	100.0	
Cefu Gaseupgi Salgyunje	49	60.5	30	37.0	2	2.5	81	100.0	
Others	116	87.9	3	2.3	13	9.8	132	100.0	
Unknown	252	93.0	7	2.6	12	4.4	271	100.0	
Chemical type									
PHMG	3642	88.0	395	9.5	103	2.5	4140	100.0	< 0.01
CMIT/MIT	611	91.6	35	5.2	21	3.1	667	100.0	
PGH	50	60.2	31	37.3	2	2.4	83	100.0	
Others	73	91.3	0	0.0	7	8.8	80	100.0	
Unknown	256	93.1	7	2.5	12	4.4	275	100.0	

A cross-tabulation of the clinical class and exposure rating is shown in Table 3. There was a significant difference between the exposure ratings and clinical classes (*p* < 0.05). Focusing on the exposure level, 365 subjects with clinical class 1, exposure rating 3 and 4, and clinical class 2, exposure rating 4, were assumed as the overestimation group. However, 344 subjects with clinical classes 3 and 4, exposure rating 1, and clinical class 4, exposure rating 2, were assumed as the underestimation group.

**Table 3:** Cross-tabulation between clinical classes and exposure ratings

		Clinical class				Total	*p*-value
		Class 1	Class 2	Class 3	Class 4		
Indoor concentration	Rating 1	2406	172	**134**	**157**	2869	0.04
	Rating 2	165	12	10	**19**	206	
	Rating 3	**137**	16	7	13	173	
	Rating 4	**169**	**9**	8	22	208	
Cumulative exposure time	Rating 1	2339	186	**144**	**200**	2869	< 0.01
	Rating 2	188	7	6	**5**	206	
	Rating 3	**159**	6	5	3	173	
	Rating 4	**191**	**10**	4	3	208	
Exposure level	Rating 1	2357	181	**143**	**188**	2869	< 0.01
	Rating 2	166	15	12	**13**	206	
	Rating 3	**162**	2	0	9	173	
	Rating 4	**192**	**11**	4	1	208	
	Total	2877	209	159	211	3456	

The demographic and exposure characteristics of these groups are shown in Table 4. The sex ratio was similar in the overestimation and underestimation groups. In the overestimation group, 90 subjects (24.7%) were under the age of 10, followed by 52 subjects (14.2%) in their 50s. In the underestimation group, 195 subjects (56.7%) were under the age of 10, followed by 80 subjects (23.3%) in their 30s. There were no current smokers in the underestimation group. The amount sprayed, the distance between the humidifier and the respiratory organ of the subject such as the nose and mouth, and spraying direction were crucial factors affecting exposure. The overestimation group was characterized by more vigorous spray amounts, closer distance to the humidifier, and direct spraying to the breathing zone than the underestimation group.

**Table 4:** Comparison of demographic and exposure characteristics in the underestimation and overestimation groups

Variables		Overestimation group		Underestimation group		Others (true-assumed group)	
		N	%	N	%	N	%
Sex	Male	193	52.9	150	43.6	1416	51.5
	Female	172	47.1	194	56.4	1329	48.4
	Unknown	0	0.0	0	0.0	2	0.1
Age at damage (years)	< 10	90	24.7	195	56.7	790	28.8
	10s	12	3.3	3	0.9	71	2.6
	20s	22	6.0	16	4.7	202	7.4
	30s	41	11.2	80	23.3	535	19.5
	40s	45	12.3	13	3.8	272	9.9
	50s	52	14.2	12	3.5	290	10.6
	60s	43	11.8	9	2.6	263	9.6
	≥70	33	9.0	3	0.9	148	5.4
	Unknown	27	7.4	13	3.8	176	6.4
Smoking status	Current smoker	17	4.7	0	0	109	4.0
	Former smoker	88	24.1	17	4.9	511	18.6
	Never smoker	260	71.2	322	93.6	2110	76.8
	Unknown	0	0	5	1.5	17	0.6
Survival status	Survival	276	75.6	208	60.5	2300	83.7
	Death	89	24.4	136	39.5	447	16.3
Job status	Employed	147	40.3	74	21.5	1165	42.4
	Unemployed	216	59.2	193	56.1	1432	52.1
	Unknown	2	0.5	77	22.4	150	5.5
Ventilation status	Ventilated	214	58.6	245	71.2	1192	61.2
	Non-ventilated	147	40.3	90	26.2	590	30.3
	Unknown	4	1.1	9	2.6	165	6.0
Sprayed amount	Least	2	0.5	18	5.2	35	1.3
	Slightly	10	2.7	38	11.1	203	7.4
	Moderate	169	46.3	180	52.3	1461	53.2
	Strong	173	47.4	99	28.8	994	36.2
	Unknown	11	3.0	9	2.6	54	2.0
Disance between the humidifier and the face of subject	< 0.5 m	131	35.9	51	14.8	752	27.4
	0.5 m ≤ − < 1 m	156	42.7	116	33.7	1052	38.3
	1 m ≤ − < 2 m	62	17.0	120	34.9	728	26.5
	≥ 2 m	14	3.8	56	16.3	207	7.5
	Unknown	2	0.5	1	0.3	8	0.3
Spraying direction	To breathing zone	278	76.2	173	50.3	1841	67.0
	To others	82	22.4	151	43.9	857	31.2
	Unknown	5	1.4	20	5.8	49	1.8

A comparison of the HD usage characteristics between the overestimation and underestimation groups is shown in Table 5. The overestimation group showed much higher usage characteristics than the underestimation group. The overestimation group showed a significant difference with other groups in the number of months used, one-time use, and daily use time.

**Table 5:** Comparison of factors affecting exposure levels in the underestimation and overestimation groups

Variables	Overestimation group		Underestimation group		Others (true-assumed group)	
	Mean	S.D.	Mean	S.D.	Mean	S.D.
Period (month)	69.8	40.1	15.4	14.3	25.0	21.6
Amount of use (mL)	27.5	32.2	14.8	8.7	14.6	12.2
Daily usage time (hr)	18.4	5.8	12.8	5.1	12.2	5.7
Usage time for sleeping (hr)	9.0	3.3	7.7	1.7	7.8	3.2
Indoor concentration (μg/m^3^)	1914.1	2401.2	771.6	665.5	647.0	690.7
Cumulative exposure time (hr)	34,548.6	22,997.0	5191.7	5667.3	7702.4	8185.3
Exposure level (μg/m^3^·hr)	49,483,924	74,191,015	3,552,275	3,657,695	4,473,117	5,260,741

* *S.D.* standard deviation

**Fig. 1:**
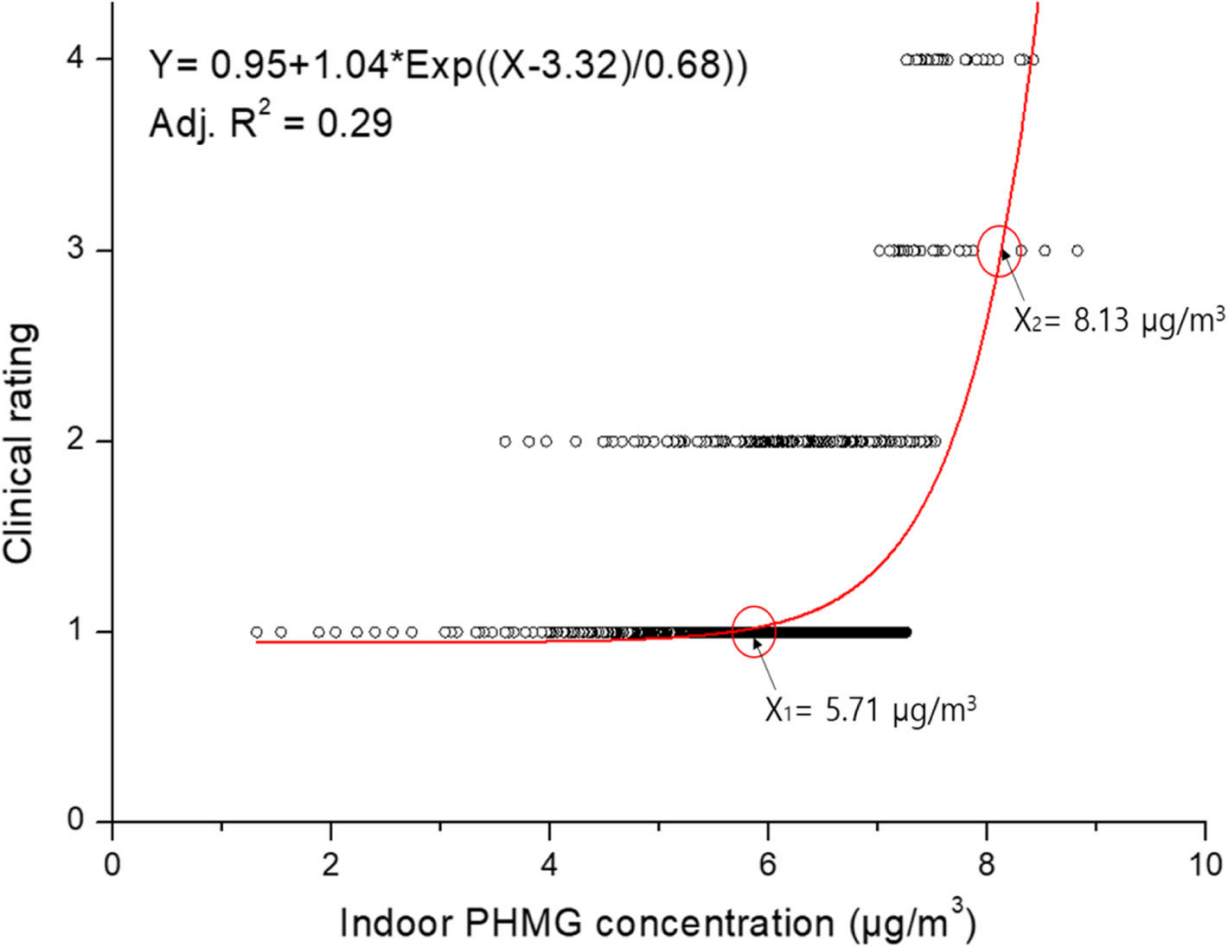
Regression analysis between indoor polyhexamethylene guanidine (PHMG) concentrations and clinical class

**Fig. 2:**
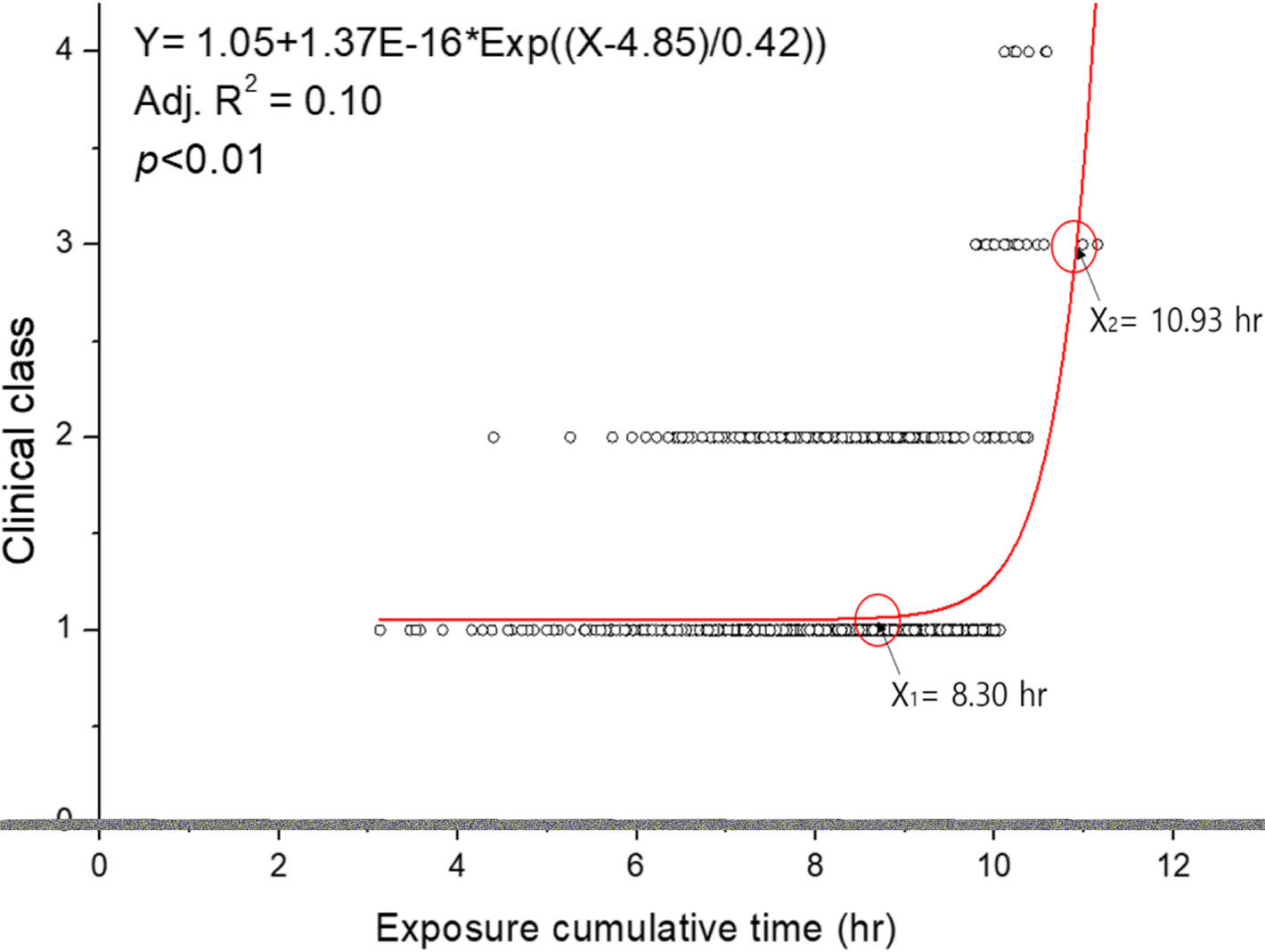
Regression analysis between exposure cumulative time and clinical class

**Fig. 3:**
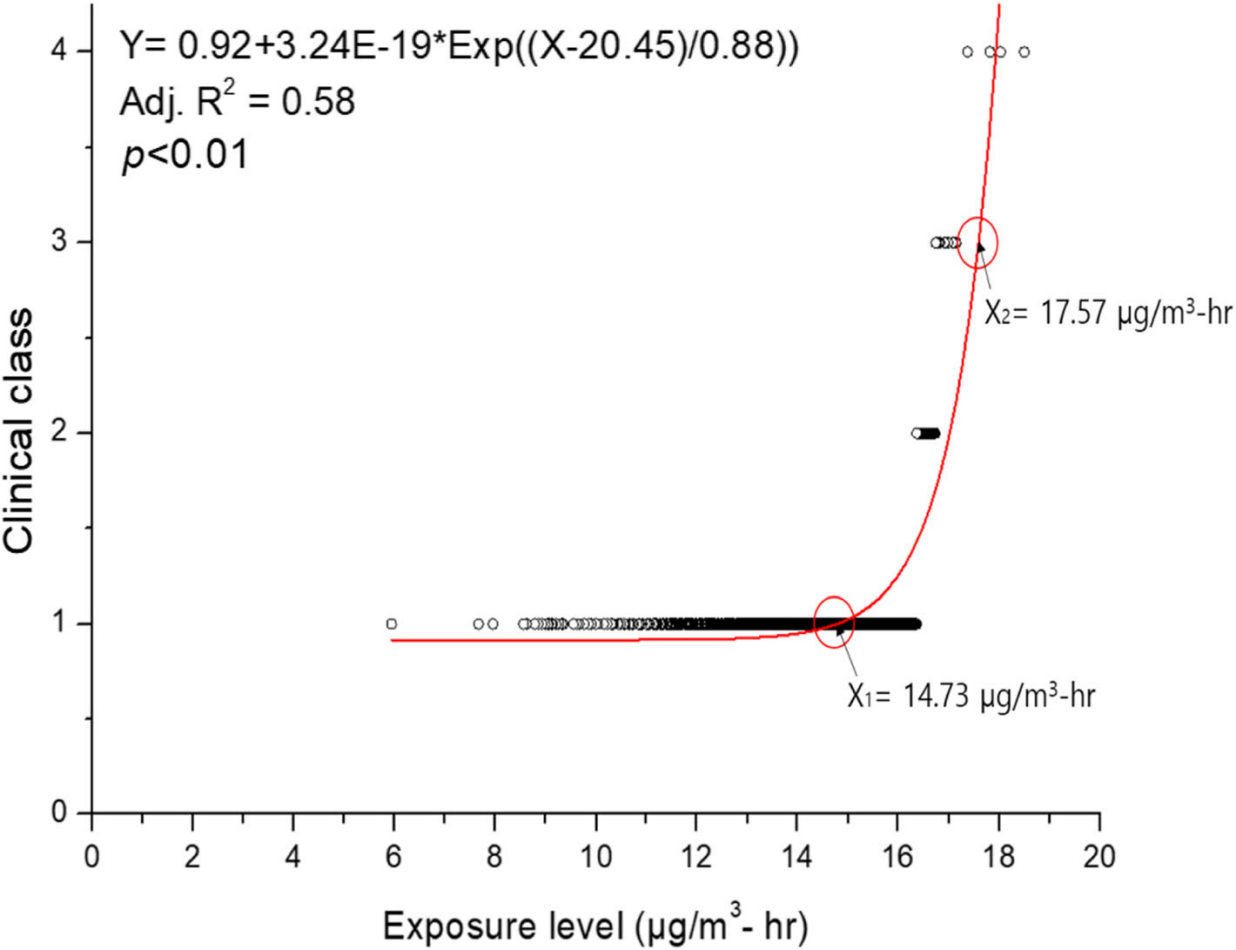
Regression analysis between exposure level and clinical class

Results of regression analysis and curve fitting of the indoor concentration, cumulative exposure time, and exposure level with the clinical classes are shown in Figs. 1, 2, and 3, respectively, after the overestimation and underestimation groups were removed. The regression formula between the exposure level (X) and clinical class (Y) was Y = 0.92 + 3.24E-19*Exp ((X-20.45)/0.88), and the coefficient of determination (R^2^) value was 0.58 (*p* < 0.01). As a result of comparing the relationship between exposure ratings and clinical classes, the breakpoint of exposure level between clinical classes 1 (unlikely) and 2 (possible) was 14.73 μg/m^3^·hr., which was considered to exert adverse health effects, especially lung disease. The exposure level for government compensation of clinical classes 3 and 4 was about 17.57 μg/m^3^·hr.

## Discussion

Exposure classification using a questionnaire might not correlate with the clinical severity classes determined through clinical diagnosis due to misclassification of exposure assessment due to erroneous statements based on recall bias [21].. This study identified the sociodemographic and exposure characteristics of subjects affected by HDs focusing on PHMG and suggested a questionnaire-based exposure assessment methodology to overcome the limitations of past exposure assessment. By identifying the limitations of the questionnaire, the exposure assessment to the humidifier disinfectant using the questionnaire can be enhanced.

Exposure assessment using a questionnaire might result in recall bias. Certain groups of subjects, such as children and the elderly, are more susceptible to exposure than the general population, and they may easily have health effects even at low exposure to hazardous agents [22]. Potential causes of increased susceptibility include genetic factors, age, sex, pre-existing disease, inadequate nutrition, and stress [23–25]. Pregnancy may also be one of the susceptible factors [26].

Although the total number of men was higher than that of women, relatively more women were in clinical classes 1 and 2, for which government compensation was recognized. The incidence of disease by exposure to hazardous pollutants is generally higher in women than in men [27, 28]. The survival rate was relatively low, given that there were 1218 deaths and 4027 survivors. However, 261 survivors and 207 deaths accounted for a high rate of adverse health effects due to HDs in clinical classes 1 and 2.

Exposure assessment of HDs was carried out using a questionnaire. Although the face-to-face interview survey consumed a great amount of effort and time, exposure classification using a questionnaire might not correlate with the clinical severity classes determined through clinical diagnosis. There was some uncertainty regarding the overestimation and underestimation exposure groups compared to clinical classes based on the clinical diagnosis. This may have been due to misclassification in the exposure assessment due to erroneous statements based on recall bias.

Relatively, there were more males in the overestimation group and more females in the underestimation group. The mortality rate was higher in the underestimation groups. This could indicate that the overestimation groups may have already recovered with no ongoing symptoms [29]. The psychological anxiety and shock from the HD disaster could also have caused overreaction [30–32]. However, the underestimation group had shorter HD usage (months) in terms of period and amount. Relatively high death rates and surrogate responses for children under 10 years old may have resulted in inaccurate exposure.

In the previous study conducted by Park et al., the HD exposure classification was conducted by setting eight influencing factors and assessing them as “yes” or “no” depending on the clinical results [33]. However, this method may further increase exposure bias because it contains subjective qualitative evaluation. Therefore, the exposure level was calculated using the indoor PHMG concentration by modeling and exposure time in this study because there is no the gold standard for observational data to verify self-reported data [34]. Apart from exposure assessment, the clinical classes based on the clinical diagnosis assuming these values are true were classified because HD-specificity in the X-ray image might be regarded as actual exposure [35].

The point of departure (POD) of the no-observed-adverse-effect level (NOAEL) value for PHMG was found to be lower than 0.15 mg/m^3^ in a 28-day inhalation toxicity test using rats [36]. The inhalation RfC value of PHMG was calculated to be 0.143 μg/m^3^ (150/1048.95) based on uncertainty correction (300; interspecies 3, intraspecies 10, duration 10, severity 1, modifying factor 1) and assessment factors (0.286, adjustment 0.179, equivalent 1.6) [37]. In this study, the exposure concentration calculated using the relationship between the indoor concentration and clinical class was about 4.7 μg/m^3^, which was approximately 30 times the difference compared to the derived RfC. This suggests that the exposure-response relationship could be obtained by using a questionnaire compared to RfC induced by animal experiments.

## Conclusions

Since recall bias and various other causes such as recovery of injury and psychological anxiety, exposure assessment to HDs using a questionnaire might not correlate with adverse health effects However, the importance of using questionnaires for past exposure assessments has increased for identifying people who may have been exposed to HDs. To overcome these limitations, the exposure levels were classified according to the clinical class. The overestimation and underestimation groups in the cross-tabulation between exposure ratings and clinical diagnosis classes were analyzed to characterize the causes of misclassification. The overestimation group may have already recovered and responded excessively because of psychological anxiety. However, relatively high mortality rates and surrogate responses observed in the underestimation group, especially under 10 years of age, may have resulted in inaccurate exposure.

## Supplementary Information

**Additional file 1.** Humidifier Disinfectants (HDs) Exposure Survey.

## Data Availability

The data that support the findings of this study are available from the corresponding author upon reasonable request.

## Abbreviations

HDs: Humidifier disinfectants
KCDC: Korea centers for disease control and prevention
PHMG: Polyhexamethylene guanidine
PGH: Oligo (2-) ethoxyethoxyethyl guanidine chloride
CMIT: Chloromethylisothiazolinone
MIT: Methylisothiazolinone
EPA: Environmental protection agency
R^2^: Coefficient of determination
POD: Point of departure
NOAEL: No-observed-adverse-effect level
RfC: Reference concentration
