# Supplementary material for: Misclassification and characterization of exposure to humidifier disinfectants using a questionnaire
Source: BMC Public Health. 2021 Jul 27;21:1458. doi: 10.1186/s12889-021-11459-4 (PMC8317285; doi:10.1186/s12889-021-11459-4)
Supplement: Supplementary file 1 — Additional file 1. Humidifier Disinfectants (HDs) Exposure Survey. [file 12889_2021_11459_MOESM1_ESM.docx]

**Humidifier Disinfectants (HDs) Exposure Survey**

| Date | (dd/mm/yy) | Surveyor |  |
| --- | --- | --- | --- |
| Applicant |  | Home  address |  |

**1. Information of family members**

| No. | Name | Smoking status  ① Current smoker  ② Former smoker  ③ Never smoker | Date of birth | Job classification | Survival status  (dd/mm/yy)  ① Survival  ② Death  The day of damage ____  The day of death _____ |
| --- | --- | --- | --- | --- | --- |
|  | Sex  ① Male  ② Female |  |  |  |  |
| 1 |  | ① ② ③ |  |  | ① Survival  ② Death  The day of damage ____  The day of death _____ |
|  |  |  |  |  |  |
|  | 1. ② |  |  |  |  |
| 2 |  | ① ② ③ |  |  | ① Survival  ② Death  The day of damage ____  The day of death _____ |
|  |  |  |  |  |  |
|  | 1. ② |  |  |  |  |
| 3 |  | ① ② ③ |  |  | ① Survival  ② Death  The day of damage ____  he day of death _____ |
|  |  |  |  |  |  |
|  | 1. ② |  |  |  |  |
| 4 |  | ① ② ③ |  |  | ① Survival  ② Death  The day of damage ____  The day of death _____ |
|  |  |  |  |  |  |
|  | 1. ② |  |  |  |  |
| 5 |  | ① ② ③ |  |  | ① Survival  ② Death  The day of damage ____  The day of death _____ |
|  |  |  |  |  |  |
|  | 1. ② |  |  |  |  |

**2. Place the humidifier disinfectants used**

| Location : ________________ (example : home, workplace, others)  Volume .: floor area ________ m^2^, height _________ m  Period of use (year-month) : - (dd/mm/yy)  Ventilation status : ① Ventilated, ② Non-ventilated, ③ Unknown |
| --- |
| (Direct drawing of indoor space and humidifier location) |

**3. Humidifier and humidifier disinfectant**

| Type of humidifier | Brand name | Usage period (dd/mm/yy - dd/mm/yy) | Strength of spray |
| --- | --- | --- | --- |
| Humidifier 1 |  | - | 1. Least 2. Slight 3. Moderate 4. Strong 5. Unknown |
| Humidifier 2 |  | - | ① Least   1. Slight 2. Moderate 3. Strong 4. Unknown |
| Type of HDs | Brand name | Usage period (dd/mm/yy - dd/mm/yy) | Daily usage amount |
| Humidifier disinfectant 1 |  | - | mL |
| Humidifier disinfectant 2 |  | - | mL |
| Humidifier disinfectant 3 |  | - | mL |
| Humidifier disinfectant 4 |  | - | mL |
| Humidifier disinfectant 5 |  | - | mL |

**4. Information of exposure to humidifier disinfectants**

| Family No. ______________ (See Question 1)  Age at damage ________________ | | Daily time of use | (hr) (min) -  (hr) (min) |
| --- | --- | --- | --- |
| Weekly usage days (average)  ____________ days | Usage time during sleeping (average)  (hr) (min) | Spraying direction   1. To breathing zone 2. To others | Distance between the humidifier and the respiratory organ of subject   1. < 0.5 m 2. 0.5 m ≤-<1 m 3. 1 m ≤-<2 m 4. >2 m 5. Unknown |
| Family No. ______________ (See Question 1)  Age at damage ________________ | | Daily time of use | (hr) (min) -  (hr) (min) |
| Weekly usage days (average)  ____________ days | Usage time during sleeping (average)  (hr) (min) | Spraying direction   1. To breathing zone 2. To others | Distance between the humidifier and the respiratory organ of subject   1. < 0.5 m 2. 0.5 m ≤-<1 m 3. 1 m ≤-<2 m 4. >2 m 5. Unknown |
| Family No. ______________ (See Question 1)  Age at damage ________________ | | Daily time of use | (hr) (min) -  (hr) (min) |
| Weekly usage days (average)  ____________ days | Usage time during sleeping (average)  (hr) (min) | Spraying direction   1. To breathing zone 2. To others | Distance between the humidifier and the respiratory organ of subject   1. < 0.5 m 2. 0.5 m ≤-<1 m 3. 1 m ≤-<2 m 4. >2 m 5. Unknown |
